# Effectiveness of Equine-Assisted Activities and Therapies for Children with Autism Spectrum Disorder: An Update

**DOI:** 10.3390/children11121494

**Published:** 2024-12-08

**Authors:** Leonardo Zoccante, Sara Sabaini, Sophia Marlene Bonatti, Erika Rigotti, Camilla Lintas, Michele Marconi, Marco Zaffanello

**Affiliations:** 1 Childhood, Adolescence, Families and Family Health Center, Provincial Center for Autism Spectrum Disorders, 37122 Verona, Italy; leonardo.zoccante@aulss9.veneto.it (L.Z.); marconimichele@icloud.com (M.M.); 2 Section of Physiology and Psychology, Department of Neurosciences Biomedicine and Movement Sciences, University of Verona, 37134 Verona, Italy; sara.psy.sabaini@gmail.com (S.S.); sophiamarlene.bonatti@univr.it (S.M.B.); 3 Department of Paediatrics, Woman’s & Child’s, University Hospital of Verona, 37126 Verona, Italy; erika.rigotti@aovr.veneto.it; 4 Department of Mental Health, ULSS 9 Scaligera, 37122 Verona, Italy; camilla.lintas@aulss9.veneto.it; 5 Corte Molon—ASD Horse Valley, 37124 Verona, Italy; 6 Department of Surgery, Dentistry, Paediatrics and Gynaecology, University of Verona, 37126 Verona, Italy

**Keywords:** equine-assisted activities and therapies, autism spectrum disorder, complementary and alternative methods, parenting stress index, short form, treatment, Vineland Adaptive Behaviour Scales

## Abstract

Background/Objectives: Autism Spectrum Disorder (ASD) is a complex neurodevelopmental condition requiring personalised therapeutic approaches. This study evaluated the effectiveness of Equine-Assisted Activities and Therapies (EAATs) in 86 children with varying ASD severity levels (levels 1–3). Methods: Vineland Adaptive Behaviour Scales and the Parenting Stress Index were used. Between May 2022 and October 2023, participants completed 20 weekly sessions of 45 min each, tailored to their individual needs. Results: Children with level 3 ASD demonstrated greater challenges in communication (level 1: 67.1 ± 29.0 vs. level 3: 30.0 ± 12.6; *p* < 0.001), daily living skills (81.0 ± 26.8 vs. 42.6 ± 18.1; *p* < 0.001), and socialisation (72.2 ± 23.2 vs. 37.3 ± 14.2; *p* < 0.001). Parental distress was higher in cases of greater ASD severity. Nevertheless, significant improvements were observed across the entire cohort in daily living skills (58.3 ± 25.5 vs. 67.8 ± 29.0; *p* = 0.023), with particularly notable outcomes in children with level 1 ASD (65.7 ± 26.9 vs. 81.0 ± 26.8; *p* = 0.010). While increases in socialisation were noted among children with level 1 ASD, these were not statistically significant (*p* = 0.073). Conclusions: EAAT fosters improvements in daily living skills, particularly in children with level 1 ASD, and has a positive impact on socialisation. For children with more severe ASD, targeted interventions are required.

## 1. Introduction

Individuals with Autism Spectrum Disorder (ASD) frequently often display difficulties in social interactions, communication, and display repetitive behaviours. The pathophysiology of ASD involves various genetic, environmental, and neurobiological factors that contribute to its diverse clinical manifestations [1]. The DSM-5 (Diagnostic and Statistical Manual of Mental Disorders, Fifth Edition), categorises ASD into three severity levels based on the degree of social communication difficulties and the occurrence of restricted, repetitive patterns of behaviour [2]. These severity levels are explicitly designed to correspond to varying degrees of functional impairment: without support, deficits in social communication result in noticeable impairments (ASD level 1); social impairments are evident even with supports in place (ASD level 2), and severe discrepancies in verbal and non-verbal social communication skills cause substantial functional impairments (ASD level 3) [2]. Recent advancements in understanding and managing ASD underscore the importance of a multifaceted approach tailored to the specific needs of everyone. Treatment strategies encompass behavioural and educational therapies [3], speech and language therapy, occupational therapy [4], family support and training, pharmacological interventions [5,6] and complementary and alternative therapies [5].

Complementary and Alternative Methods such as swimming, art therapy, music therapy, and Equine-Assisted Activities and Therapies (EAATs) have proven to be highly effective in various settings [7]. EAAT has emerged as a promising intervention for children with ASD [8]. It encompasses hippotherapy, a structured therapeutic programme, and therapeutic riding, which has its roots in recreational activities.

EAAT employs a trained therapist and a therapeutic animal to deliver animal-assisted interventions (AAIs) designed to address specific developmental needs, particularly within neuro-behavioural domains [9]. The non-verbal nature of horses encourages individuals to engage using body language and other non-verbal cues, fostering improvements in communication skills. EAAT also leverages the horse’s movement, which provides rhythmic motion to the participant’s body [10]. A review has reported improvements across several domains following EAAT, including socialisation, engagement, reductions in maladaptive behaviours, and shorter reaction times in problem-solving situations [9].

These benefits, however, are not uniform across all children, as those with ASD exhibit varying degrees of psychosocial, neurocognitive, and neuromotor dysfunction depending on the severity of their condition. Consequently, their responses to EAAT also differ, highlighting the need for personalised therapeutic approaches [7].

### Aim

Given the variability in psychosocial, neurocognitive, and neuromotor dysfunction across ASD severity levels, it is essential to determine how EAAT may address these diverse challenges. This pilot study aims to explore its specific impact across ASD severity levels using targeted metrics.

## 2. Materials and Methods

### 2.1. Subjects

This study involved 86 participants, all of whom had been previously diagnosed with ASD and whose parents/guardians provided consent for their participation. The diagnosis of ASD was made in clinical settings by specialists (LZ) following standard diagnostic criteria (DSM-5 or ICD-10). In particular, the assessments were carried out by clinicians specialising in neurodevelopmental disorders with extensive experience in diagnosing and managing conditions like ASD. Assessors were not blinded to the diagnosis of the enrolled patients. Specifically, children aged 4 to 15 years with type 1, type 2, and type 3 ASD were approached for participation. All evaluations occurred in controlled clinical settings, specifically the Paediatric Clinic and the Child and Adolescent Neuropsychiatry Outpatient Clinics at the University Hospital of Verona. This approach ensured consistency and a standardised environment for all participants. Our study took place between December 2022 and November 2023.

### 2.2. Equine-Assisted Activities and Therapies

Our previous article detailed the methodology, which presented the preliminary data [7]. Specifically, from December 2022 to November 2023, EAATs were conducted for children with ASD in a recreational setting. A multidisciplinary team consisting of a veterinarian, an equine assistant, a child neuropsychiatrist, and a specialised therapist facilitated the horse–child interaction, selecting three suitable horses for the programme. Each child participated in 20 weekly sessions of 45 min, conducted individually or in pairs, with activities that progressively increased in complexity and were tailored to the child’s learning pace, in line with the national guidelines for animal-assisted interventions.

The EAAT sessions adhered to the national AAI guidelines (no. 60/CSR; 25 March 2015). Upon arrival, each child began grooming the horse, followed by ground-based activities and mounted exercises. The sessions included tasks of increasing complexity, adjusted to the child’s abilities and learning pace, irrespective of the severity levels defined by the DSM-5. Each activity was introduced verbally and demonstrated by the therapist, allowing the child to attempt it independently or, if necessary, with assistance.

### 2.3. Vineland Test

The Vineland Adaptive Behaviour Scales (VABSs) have been utilised as a diagnostic tool to assess adaptive behaviour and various developmental disabilities. These scales evaluate an individual’s daily functioning and identify deficits in adaptive behaviour across three main domains: communication, daily living skills, and socialisation. The Vineland ABSs have proven pivotal in supporting ASD diagnoses with other diagnostic methods [11,12]. Assessments were conducted at baseline and repeated during follow-up to evaluate the module’s effectiveness on social skills, autonomy, and self-awareness.

### 2.4. PSI (Parental Stress Index)-SF

The PSI-SF is a brief questionnaire designed to assess the level of stress experienced by parents [13]. The Parental Distress (PD) subscale of the PSI-SF is essential for determining the emotional and psychological strain that parents experience in their parenting roles. The Parent–Child Dysfunctional Interaction (P-CDI) subscale of the PSI-SF evaluates how parents perceive their interactions with their children. Lastly, the Difficult Child (DC) subscale of the PSI-SF assesses parents’ perceptions of their child’s temperament and behaviour [14,15].

### 2.5. Statistical Analysis

The data were transferred from the online questionnaire created with Google Forms and saved in XLS file format. These were subsequently imported into a Microsoft^®^ Excel^®^ database for Windows 11 (access date: 30 September 2021). Statistical analyses were made using SPSS version 22.0 for Windows (SPSS Inc., Chicago, IL, USA).

Data were summarised as means ± standard deviations (S.D.) and 95% confidence intervals (C.Is.). To check the normality of continuous data, a Kolmogorov–Smirnov test was conducted. ANOVA and Tukey’s post hoc tests were used to analyse continuous variables. Binary logistic regression was used to assess the effect of the treatment (or other independent variables) on changes observed between pre- and post-treatments.

A *p*-value of less than 0.05 was considered statistically significant for all the tests. Nevertheless, it is important to note that small sample sizes, potential bias, and random error can impact *p*-values. Therefore, we will also consider statistical significance for *p*-values between 0.05 and 0.1, explaining these values and any additional evidence supporting those observed [16].

### 2.6. Ethics

The study protocols (CESC 2242 and CESC 2243) were authorised by the Ethics Committee for Research of the Integrated University Hospital of Verona. Parents and guardians provided written informed consent after a thorough explanation of the study actions, possible risks, and benefits. The study protocol was permitted by the Institutional Review Board and was conducted in accordance with ethical principles, including the Declaration of Helsinki (2013) [17].

## 3. Results

**Figure 1:**
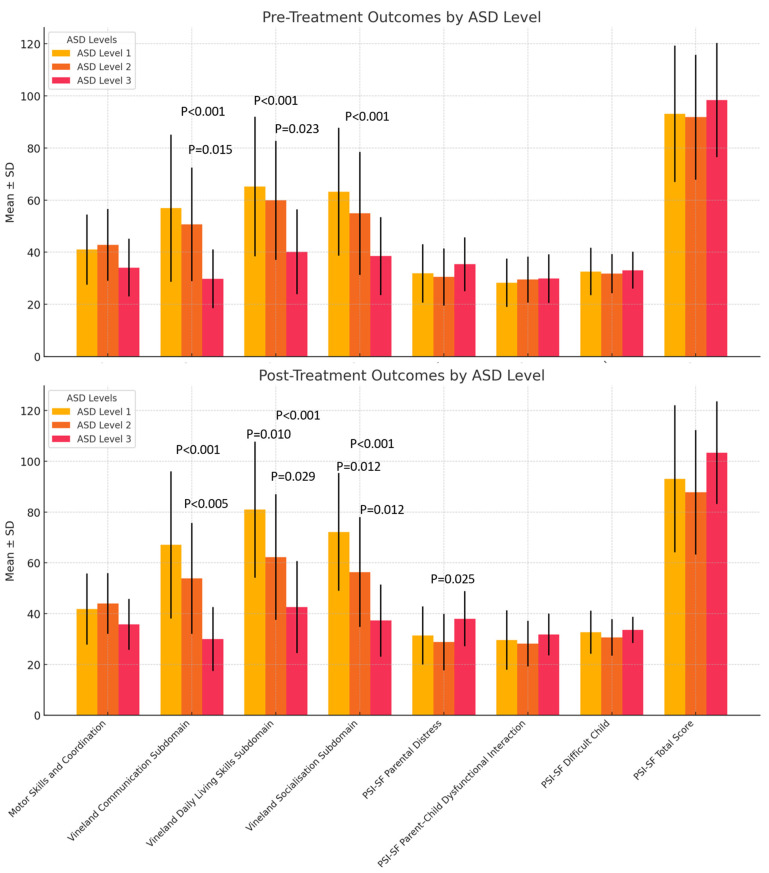
Graphical representation of the mean scores (Vineland test scores and PSI-SF scores across ASD levels) before and after treatment for the various subdomains and ASD severity levels.

The pre-treatment test results presented in Table 1 and Figure 1 highlight that communication, daily living skills, and socialisation abilities are significantly impaired with increasing severity of ASD. Communication difficulties increase with the severity of the disorder (*p* < 0.001). Specifically, post hoc comparisons reveal significant differences between level 1 and level 3 (*p* < 0.001) and between level 2 and level 3 (*p* = 0.015).

Similarly, daily living skills show a decline with increasing ASD severity (*p* = 0.001). Tukey’s comparisons indicate significant differences between level 1 and level 3 (*p* < 0.001) and between level 2 and level 3 (*p* = 0.023), suggesting that children with more severe ASD exhibit greater challenges in practical everyday skills.

In the socialisation subdomain, scores decrease as ASD severity increases (*p* = 0.001). The comparison between level 1 and level 3 is significant (*p* = 0.001), indicating that the severity of the disorder has a considerable impact on socialisation abilities.

Parental distress, assessed by the PSI-SF Parental Distress subscale, did not significantly differ between the three ASD severity levels. However, no significant changes were found for other variables analysed between the pre-treatment and post-treatment periods.

Table 2 and Figure 1 presents the post-treatment test results, highlighting that the children with more severe levels of ASD exhibit greater difficulties in communication, daily living skills, and socialisation. Furthermore, parental distress is higher for children with greater severity levels.

Mean scores at follow-up were considerably higher for level 1 participants than for those in levels 2 and 3 (*p* < 0.001). Significant differences were observed between level 1 and level 3 (*p* < 0.001) and between level 2 and level 3 (*p* = 0.005).

In the Vineland Daily Living Skills Subdomain, scores were higher for level 1 compared with levels 2 and 3 (*p* < 0.001). There were significant changes between level 1 and level 2 (*p* = 0.010), level 1 and level 3 (*p* < 0.001), and level 2 and level 3 (*p* = 0.029).

In the Vineland Socialisation Subdomain, follow-up mean scores were higher for level 1 than levels 2 and 3 (*p* < 0.001). Significant differences were found across all levels: level 1 and level 2 (*p* = 0.012), level 1 and level 3 (*p* < 0.001), and level 2 and level 3 (*p* = 0.012).

For the PSI-SF Parental Distress subscale, parental distress was significantly higher for parents of children with level 3 compared with level 2 (*p* = 0.025). However, no significant differences were found for the other variables analysed.

Table 3 shows that daily living skills improve significantly from the pre-treatment period to the post-treatment follow-up (*p* = 0.023), suggesting a positive impact of the activities on the development of these skills. However, no significant changes were observed for the other variables analysed between the pre-treatment and post-treatment periods.

Table 4 demonstrates improved communication skills in children with ASD level 1. Statistical analysis of the table reveals that, for children with level 1 ASD, there are some variations in scores between the pre-treatment and post-treatment periods. Motor and coordination skills showed no significant changes between pre- and post-treatments. A significant improvement was observed in daily living skills (*p* = 0.010). Mean scores related to socialisation increased; however, this result was not statistically significant (*p* = 0.073).

**Figure 2:**
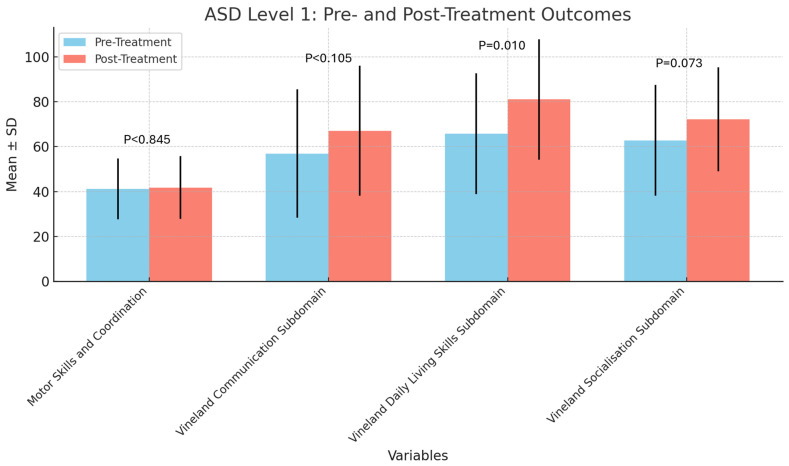
Pre-treatment versus post-treatment scores for ASD level 1 across domains. Legend: ASD, Autism Spectrum Disorder.

The bar chart in Figure 2 compares pre-treatment and post-treatment scores for level 1 ASD across different domains. The bars represent mean values with 95% confidence intervals. A significant improvement was observed in daily living skills (*p* = 0.010).

Table 5 presents the results of functional measures in a sample of children with level 2 ASD, comparing scores across various variables at the pre-treatment stage and at follow-up. There were no significant differences observed between the variables investigated.

Table 6 reports the results of functional measures in a sample of children with level 3 ASD, comparing scores across various variables at the pre-treatment and post-treatment stages. There were no significant differences observed between the variables investigated.

Table 7 demonstrates that the Vineland Daily Living Skills Subdomain has a positive and significant association with the likelihood of observing post-treatment outcomes, with a minimal increase in odds for each unit increase in this subdomain. The *p*-value of 0.036 indicates that the effect of the Vineland Daily Living Skills Subdomain is statistically significant. This finding suggests that the difference between the pre-and post-treatments was associated with changes in this subdomain. Since the Exp(B) value is 1.012, slightly above 1, it denotes a positive, albeit modest, association. These results support the notion that improvements in daily living skills may be linked to the effectiveness of the treatment.

## 4. Discussion

In the cohort of subjects with ASD included in our study, an increase in the Vineland Daily Living Skills Subdomain scores was observed. Furthermore, a significant improvement in the Vineland Daily Living Skills Subdomain and a marginal improvement in the Vineland Socialisation Subdomain were noted following treatment with EAATs in children with level 1 ASD. Finally, treatment with EAATs was associated with a statistically significant reduction in parental distress in children with level 2 ASD compared with those with level 3 ASD.

Children with ASD exhibit significant deficits in social functioning, cognitive abilities, and language skills [18]. In our study, the Vineland assessments conducted prior to treatment with EAAT revealed marked impairments in the domains of communication, daily living skills, and socialisation, which worsened with increasing ASD severity from level 1 to level 3 [2].

Communication challenges in children with ASD were significantly associated with increasing disorder severity. While significant differences were observed between levels 1 and 3, and between levels 2 and 3, on measures of communication skills, no significant differences were found between levels 1 and 2 on the Vineland Communication Subdomain or the Vineland Daily Living Skills Subdomain. Communication difficulties are a hallmark feature of ASD, manifesting in both verbal and non-verbal forms. These children frequently have difficulty starting and keeping conversations going [19,20].

Similarly, daily living skills show a significant decline with increasing severity of ASD, with statistically significant differences observed between levels 1 and 3, as well as between levels 2 and 3. Adaptive behaviours, which encompass the skills required to manage everyday tasks, are often markedly delayed in individuals with ASD [2]. While some individuals may exhibit relative strengths in daily living skills compared with their overall cognitive abilities, deficits in social skills remain prominent [21,22].

Social skills are also impaired as the severity of ASD increases, with significant differences observed between levels 1 and 3, though not between levels 1 and 2. Individuals with ASD often face challenges in socialisation [2,23], struggling to understand social cues and norms, which can hinder participation in group activities or the formation of meaningful relationships [21]. It has been reported that, of the two core diagnostic features of ASD (deficits in social communication and restricted, repetitive behaviours), social communication skills are the strongest predictors of symptom severity [24].

Parental distress in families with children diagnosed with ASD represents a significant issue, impacting both the mental health of parents and the overall family dynamic. Research suggests that parents of children with ASD, particularly mothers [25], often experience augmented levels of stress, anxiety, and depression linked to parents of neurotypical children [26]. In our study, no significant differences in parental distress, as measured by the Parenting Stress Index Short Form (PSI-SF), were observed across the three levels of ASD severity.

In the post-treatment period following EAAT, the results indicate that children with more severe forms of ASD (level 3) exhibited greater difficulties in communication, daily living skills, and socialisation. In the Daily Living Skills and Socialisation subscales, the mean follow-up scores were significantly higher for level 1 compared with levels 2 and 3. In the Vineland Communication Subdomain, no statistically significant changes were observed between levels 1 and 2 of ASD, consistent with the pre-treatment assessment. However, in the Vineland Daily Living Skills Subdomain, a statistically significant difference emerged, with level 1 scoring higher than level 2, a finding that was not present in the pre-treatment evaluation.

Previously, we reported that EAAT did not reduce parental distress and was paradoxically associated with a worsening in parental perceptions of their child’s behaviour [7]. In the current study, the PSI-SF Parental Distress subdomain showed a significantly lower score in the post-treatment period for children with level 2 ASD compared with level 3. No significant changes were observed in other PSI-SF variables analysed between the pre-and post-treatment periods.

The Daily Living Skills Subdomain evaluates essential competencies required for everyday functioning. Individuals with ASD often face challenges across various aspects of daily living skills [27,28]. Deficits in daily living skills are strongly associated with poor adult outcomes in individuals with high-functioning ASD [29]. Across the ASD cohort, EAAT demonstrated a positive impact on daily living skills. Overall, analysis of the Vineland Daily Living Skills Subdomain revealed a significant positive association with the improvements observed post-treatment. Progress in daily living skills appeared to be closely linked to the effectiveness of the intervention, with a modest but significant correlation. Notably, the greatest benefit of EAAT on daily living skills was observed in children with level 1 ASD.

In a previous study, the adaptive behaviour profile of adolescents with ASD without intellectual disability showed that daily living skills were the most deficient area, followed by socialisation and, lastly, communication [30].

It has been reported that children learn to interact with horses, therapists, and peers during sessions, promoting positive social behaviours [31]. Our study observed a mean increase in socialisation scores in children with level 1 ASD following EAAT treatment, although this increase was not statistically significant. Conversely, no statistically significant variations were found in the Vineland Socialisation Subdomain or the PSI-SF scores for children with level 2 or level 3 ASD after EAAT treatment.

Many individuals with autism experience motor and sensory challenges, which can impact their skill to perform daily living skills. Past studies have indicated that individuals with ASD may exhibit multiple motor impairments [32]. Motor difficulties are strongly associated with challenges in adaptive daily living skills among young autistic children. However, the combination of motor and sensory assessments predicts a child’s performance in daily living skills more effectively than motor or sensory evaluations conducted individually [33]. Studies have reported slight improvements in motor skills [31], although this was not observed in our study across all levels of ASD. Research has shown that sensory experiences associated with horse interaction, such as touch and smell, can enhance sensory integration and body awareness [34]. This association, however, was not examined in our study.

EAAT significantly improved irritability, hyperactivity, social cognition, social communication, and language skills (total and newly spoken words) in children with ASD. These improvements were observed from week 5 of the intervention and remained consistent even after adjusting for age and IQ [35]. Research suggests that equine therapy can lead to significant reductions in irritability, hyperactivity, and anxiety among children with ASD [34].

Parent-reported quality of life measures also showed improvement, including during the pre-treatment waiting period [34]. Conversely, another study did not report this finding [7]. In our study, no differences in parental distress levels were observed among ASD severity levels during the pre-treatment period with EAAT. However, post-treatment, a significant difference in parental distress levels emerged between parents of children with level 2 and level 3 ASD, with distress being lower in those with level 2 compared with level 3.

This study has both strengths and limitations. Among the primary limitations is the relatively small and unbalanced sample size, which may compromise the generalisability of the findings and affect statistical power. The absence of blinding among the evaluations’ assessors also introduces a potential observation bias. The wide age range of participants (4–15 years) could also influence the results, as children’s adaptive abilities vary significantly across developmental stages. Another fundamental limitation is the lack of a control group. Finally, parental stress could introduce a subjective bias in evaluating the children’s abilities.

However, the study also has several strengths. Using DSM-5 or ICD-10 diagnostic criteria, applied by experienced specialists, ensures accurate diagnostic validity for the participants. Assessments were conducted in controlled and standardised clinical environments. Using validated tools such as the Vineland Adaptive Behaviour Scales and the Parental Stress Index allowed for collecting precise and reliable measurements. The EAAT was designed following national guidelines and supported by a multidisciplinary team, thereby enhancing the credibility and effectiveness of the approach.

Given the exploratory nature of this pilot study, it is crucial to recruit a larger cohort of children, determine the most appropriate age range, and adjust the intervention according to the severity of the disorder to enhance the outcomes of EAAT. Furthermore, the long-term efficacy of EAAT should be assessed in a larger and more representative population of children with ASD.

## 5. Conclusions

The Vineland Daily Living Skills Subdomain shows a significant association with the improvements observed in the post-treatment period, albeit of modest magnitude. Following EAAT treatment, significant improvements are observed in daily living skills, particularly in children with level 1 ASD, supporting the treatment’s effectiveness in this domain. Although average socialisation scores increase for children with level 1 ASD, these do not reach statistical significance. In contrast, no significant differences are found in the skills analysed for levels 2 and 3, suggesting that the severity of the disorder may limit the treatment’s impact. A notable observation concerns parental distress, which is higher in parents of children with level 3 ASD compared with those with lower levels. Overall, the results emphasise that children with level 1 ASD benefit most from the treatment, with improvements in practical skills and a potential increase in socialisation. At the same time, challenges related to greater severity require more targeted interventions.

## Figures and Tables

**Table 1:** The table presents data on the means ± standard deviations (S.Ds.) and 95% confidence intervals (C.Is.) for the psychometric and behavioural variables collected prior to treatment across three groups of children (ASD levels 1, 2, and 3).

Variable (Pre-Treatment)	Mean ± S.D. (ASD Level 1)	95% C.I.	Mean ± S.D. (ASD Level 2)	95% C.I.	Mean ± S.D. (ASD Level 3)	95% C.I.	ANOVA One Way, *p*-Value	Multiple HSD Comparisons by Tukey
N. (%)	43 (50)		24 (27.9)		19 (22.1)			
Males n. (%)	10 (23.3)		9 (37.5)		6 (31.6)			
Age (Years)	9.4 ± 2.1	8.7–10.8	8.9 ± 2.4	7.9–9.9	8.9 ± 2.0	8.0–9.9	0.604	N.S.
Motor Skills and Coordination	41.0 ± 13.5	36.9–45.1	42.8 ± 13.8	36.9–48.6	34.1 ± 11.1	28.8–39.5	0.079	N.S.
Vineland Communication Subdomain *	56.9 ± 28.2	48.3–65.4	50.7 ± 21.8	41.5–59.9	29.8 ± 11.2	24.4–35.2	<0.001	Level 1 vs. 3: *p* < 0.001; level 2 vs. 3: *p* = 0.015
Vineland Daily Living Skills Subdomain	65.2 ± 26.8	57.1–73.4	59.9 ± 22.8	50.2–69.5	40.2 ± 16.3	32.3–48.0	0.001	Level 1 vs. 3 *p* < 0.001; level 2 vs. 3: *p* = 0.023
Vineland Socialisation Subdomain	63.2 ± 24.5	55.7–70.6	54.9 ± 23.6	44.9–64.9	38.5 ± 14.9	31.3–45.7	0.001	Level 1 vs. 3: *p*= 0.001
PSI-SF Parental Distress	31.9 ± 11.2	28.5–35.3	30.5 ± 10.9	26.0–35.1	35.4 ± 10.3	30.4–40.4	0.350	N.S.
PSI-SF Parent–Child Dysfunctional Interaction	28.3 ± 9.3	25.5–31.1	29.5 ± 8.8	25.7–33.2	29.9 ± 9.3	25.4–34.4	0.821	N.S.
PSI-SF Difficult Child	32.6 ± 9.1	29.8–35.4	31.8 ± 7.5	28.6–34.9	33.1 ± 7.1	29.7–36.5	0.851	N.S.
PSI-SF Total Score	93.1 ± 26.1	85.1–101.0	91.8 ± 24.0	81.6–101.9	98.4 ± 21.9	87.8–108.9	0.669	N.S.

Legend: C.I., confidence interval; NS, not significant; PSI-SF, Parenting Stress Index Short Form. * Single-sample Kolmogorov–Smirnov test: non-normal distribution.

**Table 2:** The table presents data on the means ± standard deviations (S.Ds.) and 95% confidence intervals (C.Is.) for psychometric and behavioural variables at the end of treatment, collected across three groups of children (ASD levels 1, 2, and 3).

Variable (Post-Treatment)	Mean ± S.D. (ASD Level 1)	95% C.I.	Mean ± S.D. (ASD Level 2)	95% C.I.	Mean ± S.D. (ASD Level 3)	95% C.I.	ANOVA One Way, *p*-Value	Multiple HSD Comparisons by Tukey
Age (Years)	9.6 ± 2.1	8.9–10.2	9.1 ± 2.3	8.1–10.1	9.2 ± 1.9	8.3–10.1	0.645	N.S.
Motor Skills and Coordination	41.8 ± 14.0	37.5–46.1	44.0 (12.0	38.9–49.0	35.8 (10.0	31.1–40.6	0.105	N.S.
Vineland Communication Subdomain *	67.1 ± 29.0	58.1–76.0	53.9 ± 21.8	44.7–63.1	30.0 ± 12.6	23.9–36.0	<0.001	Level 1 vs. 3: *p* < 0.001; level 2 vs. 3: *p* = 0.005
Vineland Daily Living Skills Subdomain	81.0 ± 26.8	72.8–89.3	62.3 ± 24.7	51.9–72.7	42.6 ± 18.1	33.9–51.3	<0.001	Level 1 vs. 2: *p* = 0.010; level 1 vs. 3: *p* < 0.001; level 2 vs. 3: *p* = 0.029
Vineland Socialisation Subdomain	72.2 ± 23.2	65.1–79.4	56.4 ± 21.7	47.2–65.5	37.3 ± 14.2	30.4–44.1	<0.001	Level 1 vs. 2: *p* = 0.012; level 1 vs. 3: *p* < 0.001; level 2 vs. 3: *p* = 0.012
PSI-SF Parental Distress	31.4 ± 11.5	28.0–35.0	28.8 ± 11.1	24.1–33.5	38.0 ± 10.9	32.7–43.2	0.029	Level 2 vs. 3: *p* = 0.025
PSI-SF Parent–Child Dysfunctional Interaction *	29.6 ± 11.7	26.0–33.2	28.2 ± 9.0	24.4–32.0	31.8 ± 8.2	27.9–35.8	0.518	N.S.
PSI-SF Difficult Child	32.7 ± 8.5	30.1–35.4	30.6 ± 7.2	27.5–33.6	33.6 ± 5.1	31.1–36.0	0.380	N.S.
PSI-SF Total Score	93.1 ± 28.9	84.2–102.0	87.8 ± 24.5	77.4–98.1	103.4 ± 20.2	93.6–113.1	0.149	N.S.

Legend: C.I., confidence interval; N.S., not significant; PSI-SF, Parenting Stress Index Short Form; S.D., standard deviation; * Single-sample Kolmogorov–Smirnov test: non-normal distribution.

**Table 3:** Descriptive table of functionality levels for the total patient cohort (*n* = 87), comparing pre-treatment and post-treatment EAAT outcomes.

Variable	Mean ± S.D. (Pre-Treatment)	95% C.I.	Mean ± S.D. (Post-Treatment)	95% C.I.	ANOVA One Way, *p*-Value
Age (Years)	9.0 ± 2.2	8.6–9.5	9.3 ± 2.2	8.8–9.8	0.472
Motor Skills and Coordination	40.0 ± 13.4	37.1–42.8	41.0 ± 12.8	38.3–43.7	0.607
Vineland Communication Subdomain	49.2 ± 25.8	43.8–54.7	55.3 ± 28.0	49.4–61.3	0.138
Vineland Daily Living Skills Subdomain	58.3 ± 25.5	52.8–63.7	67.8 ± 29.0	61.6–74.0	0.023
Vineland Socialisation Subdomain	55.5 ± 24.3	50.3–60.7	60.2 ± 25.0	54.9–65.5	0.210
PSI-SF Parental Distress	32.3 ± 10.9	29.9–34.6	31.9 ± 11.7	29.4–34.4	0.831
PSI-SF Dysfunctional Parent–Child interaction	29.0 ± 9.1	27.0–30.9	29.6 ± 10.3	27.4–31.8	0.673
PSI-SF Difficult Child	32.5 ± 8.2	30.7–34.2	32.2 ± 7.6	30.6–33.8	0.810
PSI-SF Total Score	93.9 ± 24.5	88.6–99.1	93.4 ± 26.5	87.8–99.1	0.910

Legend: C.I., confidence interval; PSI-SF, Parenting Stress Index Short Form; S.D., standard deviation.

**Table 4:** The table shows the results of the measures of function in a sample of children (n.43), comparing the scores obtained in the different variables at the pre-treatment and post-treatment levels.

ASD Level 1	Mean ± S.D. (Pre-Treatment)	95% C.I. (Pre-Treatment)	Mean ± S.D. (Post-Treatment)	95% C.I. (Post-Treatment)	ANOVA One Way, *p*-Value
Age (Years)	9.4 ± 2.1	8.7–10.0	9.6 ± 2.1	8.9–10.2	0.619
Motor Skills and Coordination	41.2 ± 13.6	37.0–45.4	41.8 ± 14.0	37.5–46.1	0.845
Vineland Communication Subdomain	56.9 ± 28.6	48.1–65.7	67.1 ± 29.0	58.1–76.0	0.105
Vineland Daily Living Skills Subdomain	65.7 ± 26.9	57.4–74.0	81.0 ± 26.8	72.5–89.3	0.010
Vineland Socialisation Subdomain	62.8 ± 24.7	55.2–70.4	72.2 ± 23.2	65.1–79.4	0.073
PSI-SF Parental Distress	32.1 ± 11.3	28.6–35.5	31.4 ± 11.5	27.9–34.9	0.798
PSI-SF Dysfunctional Parent–Child Interaction	28.4 ± 9.3	25.6–31.3	29.6 ± 11.7	26.0–33.2	0.619
PSI-SF Difficult Child	32.7 ± 9.2	29.9–35.6	32.7 ± 8.5	30.1–35.4	0.990
PSI-SF Total Score	93.5 ± 26.2	85.4–101.6	93.1 ± 28.9	84.2–102.0	0.987

Legend: C.I., confidence interval; PSI-SF, Parenting Stress Index Short Form; S.D., standard deviation.

**Table 5:** The table presents the results related to functionality measures in a sample of children with level 2 ASD (*n* = 24), assessed at pre-treatment and follow-up evaluations.

ASD Level 2	Mean ± S.D. (Pre-Treatment)	95% C.I. (Pre-Treatment)	Mean ± S.D. (Post-Treatment)	95% C.I. (Post-Treatment)	ANOVA One Way, *p*-Value
Age (Years)	8.9 ± 2.4	7.9–9.9	9.1 ± 2.3	8.1–10.1	0.720
Motor Skills and Coordination	42.8 ± 13.8	36.9–48.6	44.0 ± 12.0	38.9–49.0	0.748
Vineland Communication Subdomain	50.7 ± 21.8	41.5–59.9	53.9 ± 21.8	44.7–63.1	0.608
Vineland Daily Living Skills Subdomain	59.9 ± 22.8	50.2–69.5	62.3 ± 24.7	51.9–72.7	0.727
Vineland Socialisation Subdomain	54.9 ± 23.6	44.9–64.9	56.4 ± 21.7	47.2–65.5	0.825
PSI-SF Parental Distress	30.5 ± 10.9	26.0–35.1	28.8 ± 11.1	24.1–33.5	0.575
PSI-SF Dysfunctional Parent–Child Interaction	29.5 ± 8.8	25.7–33.2	28.2 ± 9.0	24.4–32.0	0.630
PSI-SF Difficult Child	31.8 ± 7.5	28.6–34.9	30.6 ± 7.2	27.5–33.6	0.585
PSI-SF Total Score	91.8 ± 24.0	81.6–101.9	87.8 ± 24.5	77.4–98.1	0.570

Legend: C.I., confidence interval; PSI-SF, Parenting Stress Index Short Form; S.D., standard deviation.

**Table 6:** The table presents the results of functional measures in a sample of children with level 3 Autism Spectrum Disorder (ASD) (n = 19), assessed at the pre-treatment evaluation and at follow-up.

ASD Level 3	Mean ± S.D. (Pre-Treatment)	95% C.I. (Pre-Treatment)	Mean ± S.D. (Follow-Up)	95% C.I. (Follow-Up)	ANOVA One Way, *p*-Value
Age (Years)	8.9 ± 2.0	8.0–9.9	9.2 ± 1.9	8.3–10.2	0.648
Motor Skills and Coordination	34.1 ± 11.1	28.8–39.4	35.8 ± 10.0	31.1–40.6	0.615
Vineland Communication Subdomain	29.8 ± 11.2	24.4–35.2	30.0 ± 12.6	23.9–36.0	0.968
Vineland Daily Living Skills Subdomain	40.2 ± 16.3	32.3–48.0	42.6 ± 18.1	33.9–51.3	0.668
Vineland Socialisation Subdomain	38.5 ± 14.9	31.3–45.7	37.3 ± 14.2	30.4–44.1	0.799
PSI-SF Parental Distress	35.4 ± 10.3	30.4–40.4	38.0 ± 10.9	32.7–43.2	0.458
PSI-SF Parent–Child Dysfunctional Interaction	29.9 ± 9.3	25.4–34.4	31.8 ± 8.2	27.9–35.8	0.499
PSI-SF Difficult Child	33.1 ± 7.1	29.7–36.5	33.6 ± 5.1	31.1–36.0	0.813
PSI-SF Total Score	98.4 ± 21.9	87.8–108.9	103.4 ± 20.2	93.6–113.1	0.469

Legend: C.I., confidence interval; PSI-SF, Parenting Stress Index Short Form; S.D., standard deviation.

**Table 7:** Binary logistic regression analysis for the dependent variable (pre-treatment vs. follow-up).

Dependent Variable *, Pre-Treatment = 0, Post-Treatment = 1	T	E.S.	Wald	*p*-Value	Exp(B)	95% I.C. per Exp(B)
Variable included in the model						
Vineland Daily Living Skills Subdomain	0.012	0.006	4.383	0.036	1.012	1.001–1.023
Constant	−0.751	0.390	3.707	0.054	0.472	

* Dependent variable: basal 0, follow-up 1; independent variables included in the analysis: age (years), ASD level (1, 2, or 3; categorical), motor skills and coordination, Vineland Communication Subdomain, Vineland Daily Living Skills Subdomain, Vineland Socialisation Subdomain, PSI-SF Parental Distress, PSI-SF Parent–Child Dysfunctional Interaction, PSI-SF Difficult Child, PSI-SF Total Score.

## Data Availability

The data presented in this study are available on request from the corresponding author due to privacy and ethical restrictions.
